# Re-emergence of Chandipura virus infection in India

**DOI:** 10.1080/21505594.2024.2421218

**Published:** 2024-10-28

**Authors:** Naveen Kumar, Vijay P. Bondre

**Affiliations:** ICMR-National Institute of Virology, Pune, India

**Keywords:** Chandipura virus, acute fatal encephalitis, Gujarat, India

Acute viral encephalitis is a major public health concern globally. Some clinical cases of acute encephalitis in children were reported in July 2024 in the Indian state of Gujarat. Later, Chandipura virus (CHPV) was found to be associated in many of such clinical cases of acute encephalitis. The CHPV infection induces acute encephalitis. The disease is characterized by high fever, headache, fatigue, vomiting and convulsion, with a case fatality rate of over 50% [1]. The damage to nervous tissue is considered to be the result of immunopathology due to hyper secretion of cytokines [2,3]. Death generally occurs within 24–48 hours after the appearance of the clinical symptoms [1].

CHPV belongs to the family *Rhabdoviridae* under the genus *Vesiculovirus* [2]. It was discovered in 1965 at the National Institute of Virology (NIV), Pune (India). It was found accidentally while investigating patients suffering from fever in Chandipura village in Maharashtra for dengue and chikungunya virus aetiology, therefore named Chandipura virus [4]. The major epidemic due to Chandipura virus infection occurred in 2003, wherein the deaths of over 300 children were reported in Maharashtra and Andhra Pradesh states. Although the region has become endemic for Chandipura virus, there has been a drastic reduction in the cases over the years since 2003–2004 [2]. Only sporadic cases have been reported since 2010–2011 [2]. This year, the first positive case was detected on 14 July 2024 and since then, over 20 encephalitis-associated deaths have been suspected due to Chandipura virus infection in the Indian state of Gujarat [5] and Rajasthan [6].

The NIV, which has been a central referral laboratory for testing, has received samples from 70 cases of encephalitis, out of which 14 have been confirmed positive for CHPV; 11 by detection of CHPV genome in serum and/or cerebrospinal fluid (CSF) by polymerase chain reaction (PCR) and 3 by detection of virus-specific IgM antibodies. The samples were also tested negative for Japanese encephalitis virus, enterovirus, dengue virus, Chikugunya virus, Zika virus, Crimean-Congo haemorrhagic fever virus, herpes simplex virus 1 (HSV) and HSV-2, human herpes virus 1 (HHV-1), HHV-2, HHV-6, HHV-7, Rickettsia, Scrub typhus and *Orientia tsutsugamushi*. The positive cases belonged to Mehsana, Aravali, Gandhinagar, Rajkot, Kheda, Ahmedabad, Vadodara and Jamnagar districts of Gujarat. All the positive samples belonged to children aged 1–15 years.

Sera from 17 animals, comprising six goats, six cows, four buffaloes, and one horse, out of a total of 432 incontact animal samples, reacted against CHPV. However, some of these CHPV-positive sera, together with some negative sera, also reacted against the rabies virus. Therefore, at this moment it could not be ascertained that the antibodies are specifically directed against CHPV. This needs further in-depth investigation.

So far the disease has been restricted to India [2]. A few cases have also been suspected in Sri Lanka. Only Central and South Indian states, which include Gujarat, Maharashtra, Madhya Pradesh, Bihar, Andhra Pradesh, Karnataka, Tamil Nadu, Odisha and Kerala have so far experienced the CHPV outbreaks [2]. The virus has been detected in Nigeria and Senegal in hedgehogs and sandflies, however, evidence of human infection from these countries so far is lacking.

The virus is considered to be transmitted primarily through sand flies, particularly those belonging to the genus *Phlebotomus*, especially *Phlebotomus papatasi* species [7]. The sand fly can also transmit the CHPV by venereal route [8], suggesting a possible role of the male fly in maintaining the CHPV activity in nature. The sand fly of the genus *Sergentomyia*, which is abundantly present in endemic areas, also known to harbour CHPV [9]. There is no evidence of direct human-to-human transmission. Animals such as pigs, buffaloes, cattle, goats and sheep have been shown to have antibodies against CHPV in the endemic areas [10]. However, the direct evidence in terms of detection of virus from the animals is lacking. The animal reservoirs, if any, that harbours CHPV are also not known, either. Therefore, the transmission of CHPV infection is not well understood.

The virus can be detected in throat swabs, serum and CSF [1] by polymerase chain reaction (PCR) and can be isolated in Vero, Madin-Darby canine kidney (MDCK) and Rhabdomyosarcoma (RD) cell lines [1]. Due to the rapid death of the affected individuals, the serological diagnosis is of limited value but IgM antibodies can be detected in some of the patients during the febrile stage. Moreover, PCR confirmed only 14 of the 70 suspected cases of acute encephalitis. Therefore, the nature of samples and appropriate time of collection from the infected patients needs to be precisely determined and needs further investigation. Besides, comorbidities should also be taken into consideration to rule out the association of the CHPV in the cases associated with acute encephalitis.

Currently, there are no approved antivirals against CHPV. In laboratory studies, favipiravir [11]) and ribavirin [12] have been shown to exert antiviral activity against CHPV. However, their clinical efficacy has not yet been proven. Effective antivirals that can restrict both virus growth and reduce immunopathology due to hypercytokinemia (inflammatory response) will be very helpful. In the past, the National Institute of Virology, Pune, India has developed two vaccine candidates, inactivated vaccine [13] and an envelope glycoprotein (G)-based recombinant protein-subunit vaccine [14]. Both of these vaccines induced a potent antibody and cell-mediated immune response and provided complete protection against virulent CHPV challenge infection in mice. However, because of very limited geographical distribution of the disease and the limited number of cases, the vaccine has not yet been commercialized.

The CHPV that belongs to the 2003 outbreak has been more virulent as compared to the virus initially isolated in 1965 or those associated in other outbreaks. The sequence analysis of CHPV from 1965 to 2007 showed an identity of >97% [15] with several functional motif characteristics of the virus conserved within all the isolates [15]. However, comprehensive genetic and antigenic characterization and pathogenicity of the current and past CHPV isolates in terms of their virulence need to be investigated in detail.

In summary, CHPV is a very poorly studied virus, very little is known about its replication, interaction of viral RNA/proteins with cellular factors, immune responses to the infection, transmission, animal reservoir, and pathogenesis, particularly the factors triggering the neuronal cell death. Likewise, how the virus suppresses the blood brain barrier and invades the central nervous system to cause encephalitis while evading host immune surveillance is not known. The current epidemic of CHPV infection warrants development of rapid diagnostic test prophylactic and therapeutic agents, besides developing early diagnostic tools. From the public health point of view, integrated vector management, which includes public awareness and the implementation of legislation to prevent mosquito breeding, is essential.

## Data Availability

No primary data (figures and tables) are included in this article.
